# Liquid Bridge-Induced SERS Hotspots: Nitrobenzene Grafting on Silver–Gold Nanoparticles

**DOI:** 10.1021/acsomega.5c04719

**Published:** 2025-07-10

**Authors:** Florian Küstner, Florian Lackner

**Affiliations:** † Institute of Physics, 27267 University of Graz, 8010 Graz, Austria; ‡ Institute of Experimental Physics, Graz University of Technology, 8010 Graz, Austria

## Abstract

The crafting process of nitrobenzene on silver@gold nanoparticles from a solution of 4-nitrobenzenediazonium-tetrafluoroborate in acetonitrile strongly depends on the silver to gold ratio. The investigation of SERS substrates consisting of gold, silver, and silver@gold nanoparticles, prepared by employing a helium droplet-based synthesis approach, provides insight into the underlying plasmon-enhancement mechanism. Gold reacts slowly with the diazonium salt; however, the reaction can be accelerated by photoexcitation at the surface plasmon resonance. In contrast, silver reacts spontaneously, which prevents plasmon-induced local grafting. UV/vis absorption spectroscopy and Raman spectroscopy indicate that, in the case of silver, SERS hotspot formation is mediated by acetonitrile liquid bridges that initially connect the nanoparticles. This provides a novel approach to simultaneously generating and selectively functionalizing SERS hotspots.

## Graphical abstract

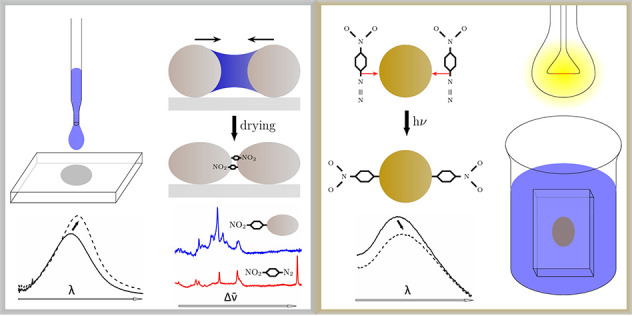

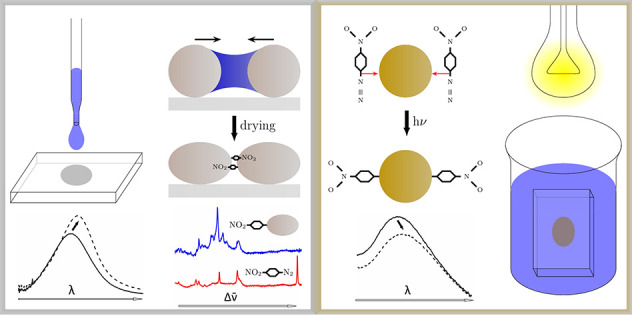

## Introduction

Raman spectroscopy has become a powerful technique with diverse applications. It is an important tool in analytical chemistry and solid-state physics, but more recently, also in biology and medicine.^1–3^ A major breakthrough in the history of Raman spectroscopy was the introduction of surface-enhanced Raman spectroscopy (SERS), which greatly improved the sensitivity of the method. This enabled the creation of SERS tags for biosensing and bioimaging.^4–7^ SERS tags consist of Raman-active organic molecules attached to metallic nanoparticles. The nanoparticles enhance the Raman signal via the SERS effect,^8^ while the Raman shift is influenced by the local molecular environment, such as, for example, different types of tissue.^3^ This dual functionality — the combination of signal enhancement and molecular sensitivity — enables SERS tags to detect target molecules using Raman spectroscopy or SERS microscopy.^4^

Typically, SERS tags consist of four material layers: A noble metal nanosubstrate below a layer of Raman reporter molecules covered by a protective layer with the target molecules on top. The metal nanosubstrate amplifies the Raman signal from the attached reporter molecules through surface-enhanced Raman scattering (SERS).^7,8^ The protective layer ensures that the reporter molecules stick to the surface of the metal nanosubstrate, and the targeting molecules provide the SERS tag with selectivity for certain biological targets. Typically, nitrogen- and sulfur-containing dyes and small molecules with thiol groups^4^ are used as Raman reporters, which are only weakly bound to the metal surface. However, there are various reports on the modification of metal surfaces with aryl diazonium salts, which results in a more stable covalent metal-carbon bond between reporter molecules and the surface.^9–14^ Thus, the surface functionalization of plasmonic nanoparticles with aryl diazonium salts is interesting for the development of more robust SERS tags due to the strong bond between reporter molecules and the metal surface.

Gold or silver nanospheres are mainly used as SERS-active substrates. Silver has the advantage of a stronger enhancement of the Raman signal; however, gold is chemically more stable and better compatible with biomaterials.^4^ Considering these aspects, nanoparticles that comprise a silver core, for exploiting the superior plasmonic enhancement of Ag, and a Au shell for improved stability and biocompatibility, potentially represent a SERS substrate that can combine the advantages of both materials.

The helium nanodroplet^15,16^ synthesis approach allows for the production of a wide variety of nanoparticles in the sub-10 nm size regime. Plasmonic nanoparticles made of copper,^17^ silver,^18–20^ and gold^21,22^ have been successfully formed. A specialty of the method is the combination of different species to form core@shell-structured particles, which has been shown for a large variety of materials,^22–27^ including metals combined with organic molecules.^28–30^ Today, helium droplet-based nanoparticle synthesis is well established and employed routinely for more than a decade.^24^ In particular, the synthesis and deposition of particles comprising silver and gold has been extensively studied and is well understood,^19,23^ also in terms of the plasmonic properties of the formed structures.^27^

The plasmon resonance of such hybrid nanostructures depends on the silver-to-gold ratio, enabling the localized surface plasmon resonance (LSPR) to be tuned to the wavelength of the Raman laser. The synthesis and characterization of such a SERS substrate is subject to this work; in particular, we explore the functionalization of Ag, Au, and Ag@Au nanoparticle substrates with an aryl diazonium salt, exploiting a grafting mechanism that has recently been discovered for diazonium salts at Au nanodiscs.^10^

Another interesting aspect of such tailored plasmonic substrates is their potential to form so-called plasmonic hotspots. The SERS effect relies on the local enhancement of the electromagnetic field in nanoscopic regions at the substrate.^8^ The field enhancement is highest at nanosized tips, edges, or small gaps between adjacent nanoparticles. These regions are associated with the highest local Raman enhancement factors, and the total detected Raman signal originates almost exclusively from these areas of the sample.^8^ In general, molecules do not naturally attach to these specific geometric features, such as tips or gaps. Therefore, it is important to develop strategies for efficiently placing molecules at plasmonic hotspots. One possibility is offered by plasmon-induced grafting, where chemical deposition processes are mediated by excited plasmon resonances. This approach has been proven for various concepts, whereby the mechanisms underlying the plasmon-induced grafting processes are controversially discussed and may differ depending on the particular application.^31^ The excited plasmon resonance is a collective electron oscillation that initially generates a very strong evanescent electromagnetic field.^32^ This oscillation subsequently decays either through the emission of a photon or through the excitation of hot charge carriers in the metal particle, which in turn can excite lattice vibrations and eventually heat up the particle.^33^ Consequently, it is possible that the observed grafting reaction is mediated by the enhanced electromagnetic field, the injection of hot charge carriers, or the increased temperature.^31^ For gold nanoparticles, a plasmon-induced grafting process of diazonium salts has been observed and tentatively attributed to a hot electron transfer.^34^ By taking advantage of the different time scales between spontaneous and the much faster plasmon-induced grafting, it is possible to stimulate the process locally at plasmonic hotspots. In comparison, grafting of molecules at hotspots near the surface of gold nanoparticles illuminated with a laser takes place within a few minutes, while spontaneous grafting onto a gold surface is a very slow process that can take several hours.^13,35^ However, less noble substrates can reduce the diazonium salts to the corresponding aryl radicals and, therefore, make spontaneous grafting highly efficient.^13^ Consequently, the functionalization of silver bulk^12^ or silver nanoparticles^36^ with aryl diazonium salts cannot be controlled by plasmon excitation.

In this work, 4-nitrobenzenediazonium-tetrafluoroborate (NBDT) was used to functionalize gold, silver, and silver@gold nanoparticles synthesized using the helium nanodroplet^15^ synthesis approach,^23,24^ a method which allows for the production of silver@gold (Ag@Au) core@shell nanoparticles with tailored plasmonic properties.^27^ Acetonitrile (AN) serves as a solvent in the functionalization process, adapting to the different substrates that have been used. The samples are analyzed by employing UV/vis absorption spectroscopy and Raman spectroscopy, which provides insight into particle rearrangement on the substrate caused by acetonitrile liquid bridges between the nanoparticles.^37,38^ A process known as evaporation-induced self-assembly of nanoparticles,^39^ which has been previously described in the context of two and three-dimensional superlattice growth.^40–42^ In the context of SERS substrates, we find that this process shows the potential to deliberately place molecules into SERS hotspots between silver nanoparticles with high efficiency.

Furthermore, the analysis of the Raman spectra provides information about how the molecules attach to nanoparticle surfaces. The grafting of NBDT on flat gold and silver surfaces has already been well investigated. For gold both, the direct binding via a Au–N bond and the dediazoniation followed by the formation of a Au–C bond in the para position to the nitro-group have been reported.^12,13^ However, for plasmonic gold nanoparticles, the formation of a Au–N bond has not been reported so far.^10^

## Experimental Section

Silver (Ag), gold (Au), and silver@gold (Ag@Au) core@shell nanoparticles are employed as substrates, synthesized and deposited on fused silica plates by using the helium nanodroplet synthesis approach.^24^ Figure 1a shows a schematic of a helium nanodroplet synthesis apparatus. The adjusted stagnation conditions for the generation of helium droplets correspond to 60 bar and 10 K, and a nozzle with a 5 μm diameter is used. Under these conditions, the estimated diameter of the helium droplets corresponds to about 80 nm with about 7 × 10^6^ He atoms per droplet.^15,29,43^ Helium nanodroplets collect individual Ag and Au atoms at the respective pickup region, which subsequently coagulate and form nanoparticles. With both pickup zones operated at the same time, core@shell nanoparticles with tailored plasmonic properties can be synthesized.^27^ The surrounding liquid helium is thereby cushioning the deposition process^44^ and, eventually, the residual helium evaporates, leaving behind the nanoparticles at the fused silica surface at high-vacuum conditions in the apparatus.^24^ Nanoparticles were deposited for two hours at each deposition spot, with a size of about 5 × 5 mm^2^. The deposition rate is measured before and after the deposition using a microbalance. Additionally, the attenuation of the helium was measured using a quadrupole mass spectrometer (QMS), which provides an additional method used to monitor the deposition process. At the experimental conditions listed in Table 1, each deposition spot is estimated to feature approximately 5 × 10^–9^ mol atoms, the surface coverage (s.c.) is estimated with about 10% and the volume ratio of gold:silver has been adjusted to 1:2 for the silver@gold particles.^27^ Similarly, silver and gold particle spots with varying surface coverages are deposited by adapting the deposition time. Figure 1b,c shows the dependence of the absorption spectra of the prepared substrates on the surface coverage.

**Figure 1:**
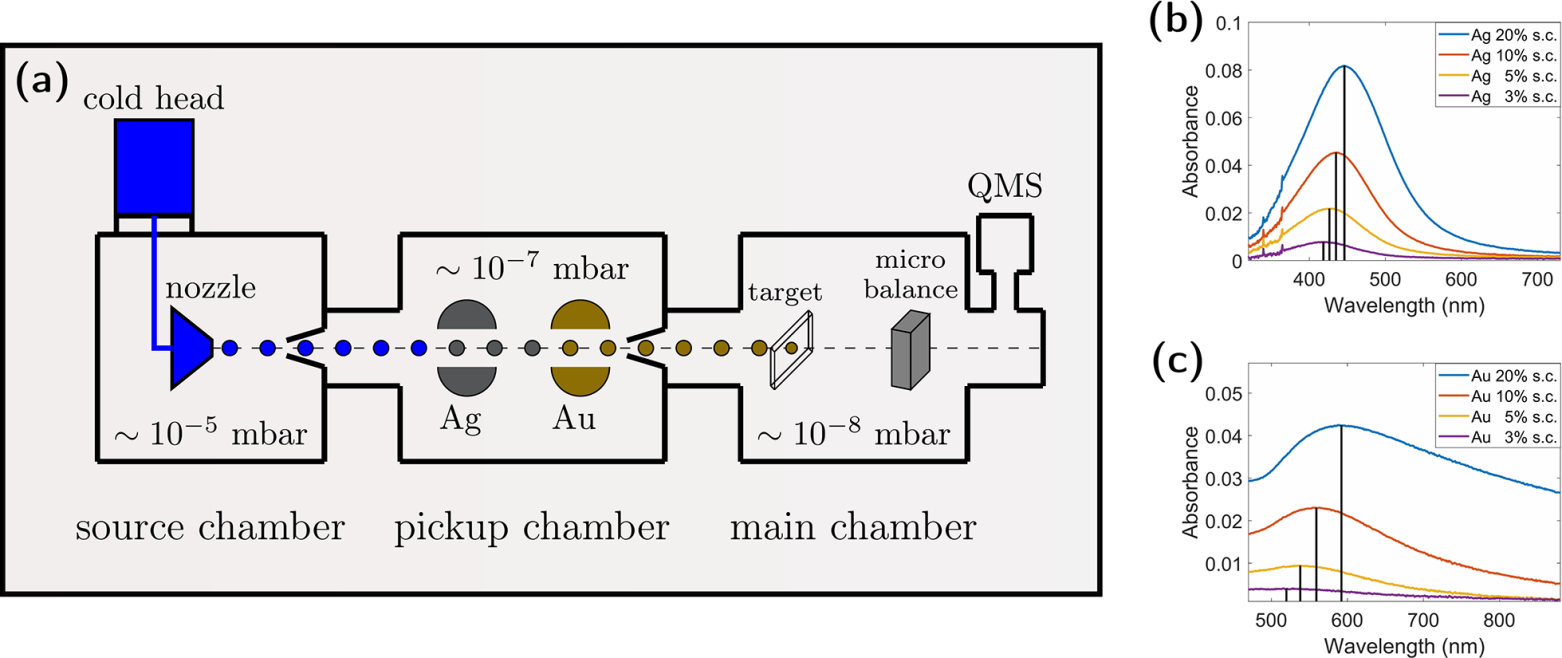
(a) Sketch of a helium nanodroplet synthesis apparatus. A quadrupole mass spectrometer (QMS) mounted off-axis is used to monitor the beam attenuation in situ. Helium under a pressure of 60 bar and a temperature of 10 K is expanded through a nozzle with a 5 μm diameter to produce droplets with a diameter of about 80 nm. They collect gold and silver Atoms in the pickup chamber that coagulate to nanoparticles inside the droplets, which are subsequently deposited on a fused silica substrate in the main chamber. (b, c) UV/vis absorption spectra of silver and gold, respectively, for varying surface coverages (s.c.).

**Table 1:** Deposition Rate and Beam Attenuation Adjusted for the Helium-Droplet-Based Synthesis of the Different Substrates

	deposition rate $\left(\frac{10^{-5}\;\mathrm{\mu g}}{{\mathrm{cm}}^{2}\;s}\right)$		attenuation (%)	
sample name	gold	silver	gold	silver
Ag		7.2		45
Au	9.2		46	
Ag@Au	4.6	4.7	19	23

To functionalize the surface of the metal nanoparticles, two different processes were applied. On one hand, the deposition spots of silver particles, which react spontaneously with NBDT, are covered by two drops of a solution of 10^–6^ mol/L NBDT (97%) in acetonitrile (≥99.9%) and left to dry up (drop procedure). On the other hand, the deposition spots with the gold particles are functionalized by dipping them into a solution of 10^–3^ mol/L NBDT in acetonitrile for 3–10 min (dip procedure). During immersion, the gold particles are illuminated by a 75 W light bulb placed 9 cm above the substrate. After removal, the sample is rinsed with acetonitrile. Both procedures have been applied to functionalize the silver@gold core@shell particles. Prior to functionalization, all substrates (gold, silver, and silver@gold) are flushed with pure acetonitrile.

UV/vis extinction spectra are recorded using a spectrophotometer UV-1800 from SHIMADZU from 300 to 900 nm. The illuminated spot size is about the size of the areas where the nanoparticles are deposited. At each step in the sample preparation process, i.e., flushing of the surface, functionalization, and rinsing with acetonitrile, a UV/vis extinction spectrum was recorded.

Raman spectra (SERS) are recorded for the functionalized plasmonic nanoparticles on the fused silica substrates. For these measurements, a homemade setup has been used. The employed Raman spectrometer consists of a PGL-FS-532 laser, of which about 5 mW is used for spectroscopy. The setup comprises an OLYMPUS MPlan N 20*X*/0.40 objective, a KYMERA-328I–B2 spectrometer, and a DU401A-BR-DD USB Camera from ANDOR. The Raman laser was guided onto the sample under normal incidence.

## Results and Discussion

The grafting of diazonium salts onto gold surfaces is a slow process, which can be accelerated by the field enhancement of localized surface plasmons of nanostructured particles, enabling photoselective functionalization in hotspots.^34^ As an electrochemically less noble element, silver reacts very quickly with diazonium salts, which prevents photoselective functionalization at plasmonic hotspots. In the following, an alternative way to preferentially localize NBDT molecules at hotspots between adjacent silver particles is presented, which takes advantage of emerging liquid bridges of acetonitrile between neighboring particles. As shown in Figure 2a, it is observed that acetonitrile, when dropped onto silver particles and dried, results in a change in the measured absorption spectrum. The redshift of the plasmon resonance peak induced by this process is comparable to the change associated with an increase in surface coverage, as shown in Figure 1b, indicating a smaller average distance between the silver particles. The numerically calculated extinction cross sections for a pair of silver particles described in the inset of Figure 1c shows that the observed peak shift and absorption increase are in very good agreement with the theoretical prediction for reduced particle distances. Note that the measured resonances are much broader than the calculated peaks since in the experiment, there is a broad distribution of particle sizes that is not captured by the calculation, which is carried out only for the mean particle size. A similar effect may be expected by a structural rearrangement from spherical to elliptical particles,^32^ which can contribute to the observed peak shift. However, this effect cannot account for an increase in the absorption. The influence of residues from acetonitrile on the particle surfaces on the spectra can be excluded because the Raman spectra of the silver particles recorded after the treatment with pure acetonitrile drops do not show any signal, although acetonitrile is a highly Raman-active molecule.

**Figure 2:**
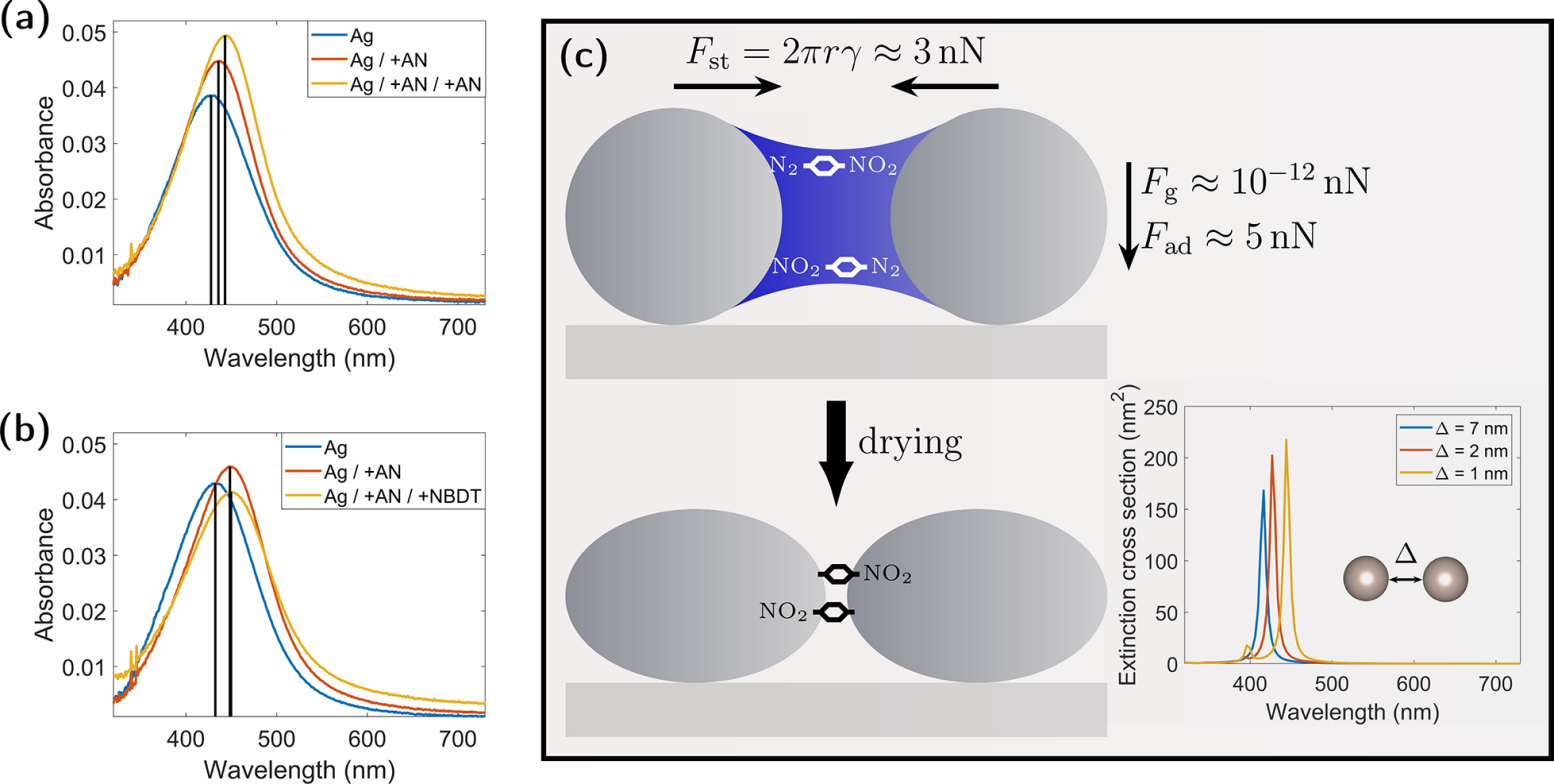
(a) UV/vis absorption spectra of a silver deposition spot (blue) and the spectra after applying and drying two drops of acetonitrile (red) and after repeating this procedure (yellow). (b) Same as (a) on a different deposition spot for the blue and red curves, while the yellow spectrum corresponds to a subsequent treatment with two drops of 10^–6^ mol/L NBDT in acetonitrile. (c) Sketch of the proposed mechanism of liquid bridges between particles, which leads to the reduction of the particle distances and thus to the observed red shift of the spectra after treatment with acetonitrile. In the figure, estimated values for the forces due to surface tension *F*_st_, gravitation *F*_g_ and adhesion *F*_ad_ are given. The inset gives numerically calculated extinction cross sections for a pair of silver particles with radii of 2 nm for varying gap distances Δ between the particles.^45^

In panel (b) of Figure 2, the yellow spectrum corresponds to the result after treating the silver nanoparticles with two drops of 10^–6^ mol/L NBDT. The applied amount of NBDT is sufficient to oxidize about 10% of the Ag atoms at the treated spot. In principle, this should lead to a blue shift of the plasmon peak due to the reduced Ag particle size. However, this effect is compensated by a red shift due to the particle rearrangement by acetonitrile and the changed environment of the plasmonic particles, as made evident by Raman measurements. Under the given conditions, these effects cancel each other, which explains the observed constant plasmon resonance peak position.

The observations described so far suggest that treatment with drops of acrylonitrile dissolved in NBDT reduces the gap between silver particles during drying. This conclusion is explained by the formation of liquid bridges between the particles during the drying process,^46^ as outlined in Figure 2c. Liquid bridges generate static and dynamic forces between the particles.^47^ It has been shown that with some approximations, a reasonable analytical expression for the description of the static force between two particles can be found.^48^ The upper limit of this force, *F*_st_, for complete wetting of two equally sized particles with radius *r* and a vapor–liquid surface tension γ is given by^48,49^

$$F_{\mathrm{st}}=2\pi r\gamma$$ 1

Assuming a particle radius of 2 nm, which is about the mean radius of the synthesized particles,^27^ and using the air-acetonitrile surface tension of about 30 mN/m,^50^ eq 1 gives a force of about 3 nN. This is strong compared to the gravitational force *F*_g_ of about 10^–12^ nN per particle. Furthermore, this value is similar to the expected adhesion force *F*_ad_ in the range of 1 nN to 10 nN per particle.^51,52^ The resulting forces are therefore in a range that allows the particles to move on the surface. Thus, the reduction of the particle gaps by liquid bridges can be explained well by the acting forces. Additionally, small silver nanoparticles show liquid-like behavior while conserving their crystalline structure.^53^ Acting forces from liquid bridges can therefore stretch the particles and reduce the distance between adjacent particles without rearranging their positions. These two processes lead to similar conclusions, and possibly both contribute to the observed peak shift. The described principles provide a possibility for the efficient direction of molecules toward plasmonic hotspots between nanoparticles because dissolved molecules concentrate at the drying liquid bridges and remain as dry residue at the interparticle gaps formed by the liquid bridges.

As shown in Figure 3, the UV/vis extinction spectra of drop-treated silver particles in panel (a) are compared with gold particles treated with the dip procedure under illumination in panel (b). It becomes evident that the position and shape of the LSPR peak for the gold particles are not affected by the rinsing procedure with acetonitrile. The same observation is made for drop-treated gold particles, demonstrating that nanoparticle reorientation with acetonitrile is different for gold and silver. After immersion in the NBDT solution under light exposure for 3 min, the peak is red-shifted by 13 nm. This shift does not change for longer treatment durations. The observed red shift can be explained by an altered dielectric environment of the gold particles due to NBDT that has attached to the gold surface.

The recorded UV/vis extinction spectra of the silver@gold core@shell particles are presented in Figure 3c,d. Panel (c) shows the results obtained for the dropwise treatment using the diluted reagent, as described above for the silver particles. Panel (d) corresponds to the results obtained for the dip-treatment, using the procedure described for the gold particles. A striking difference with respect to the previous cases that can be readily recognized is that in this experiment, the rinsing with acetonitrile shows a weaker effect on the peak position. Consequently, it is concluded that the structural rearrangements of the particles on the surface are small compared to the treatment with acetonitrile drops. Beyond the scope of the present article, this could be subject to further experiments targeting the investigations of the behavior of the surface deposited particles themselves under the influence of liquids and drying up liquids.

In the following, the results obtained for silver@gold particles after treatment with the reagent are discussed. The experiment performed with the dip procedure shows a much stronger signal reduction as the experiment with pure gold particles, which indicates that the gold layer covers the silver core just partly at the adjusted 1:2 gold:silver ratio, and therefore, a certain part of silver can be dissolved. In contrast to experiments with pure silver particles, which fully dissolve in a 10^–3^ mol/L NBDT solution, a considerable part remains undissolved on the fused silica surface. The corresponding LSPR peak position is red-shifted. The red shift in this case is, again, explained by a combination of the effects of silver dissolution, change of the particle distribution, and change of the environment of the plasmonic particles. Therefore, the UV/vis extinction spectrum alone is not sufficient in order to get information about the surface functionalization or, in particular, the change of the dielectric environment of the plasmonic particles. However, the results provide insight into the composition of the remaining particles at the substrate surface. For complete dissolution of silver, a spectrum that resembles the yellow graph in Figure 1c with an LSPR peak at 538 nm would be expected. However, the peak-position of the remaining particles at the surface is located at 505 nm, which indicates a contribution from undissolved silver.

The measured Raman spectra obtained for the discussed samples are shown in Figure 3e, compared with a Raman spectrum measured on bare NBDT powder. The Raman features up to 1800 cm^–1^ of all samples, including the powder, are located at similar positions, confirming surface-attached molecules. However, the prominent N≡N stretching peak of the diazonium group in NBDT is missing in Raman measurements on metal nanoparticles, providing clear evidence that all attached molecules have undergone dediazoniation by reacting with the surfaces of the metal particles. In Table 2, the measured Raman shifts are compared with literature values for nitrobenzene molecules. The peak position for the C=C stretching mode is in perfect agreement, whereas for the C–H in-plane bending mode and for the N=O stretching mode, the measured signals exhibit a slightly stronger Raman shift, which can be interpreted by a higher binding energy or a shorter binding length due to the interaction with the surface of the nanoparticles.

**Figure 3:**
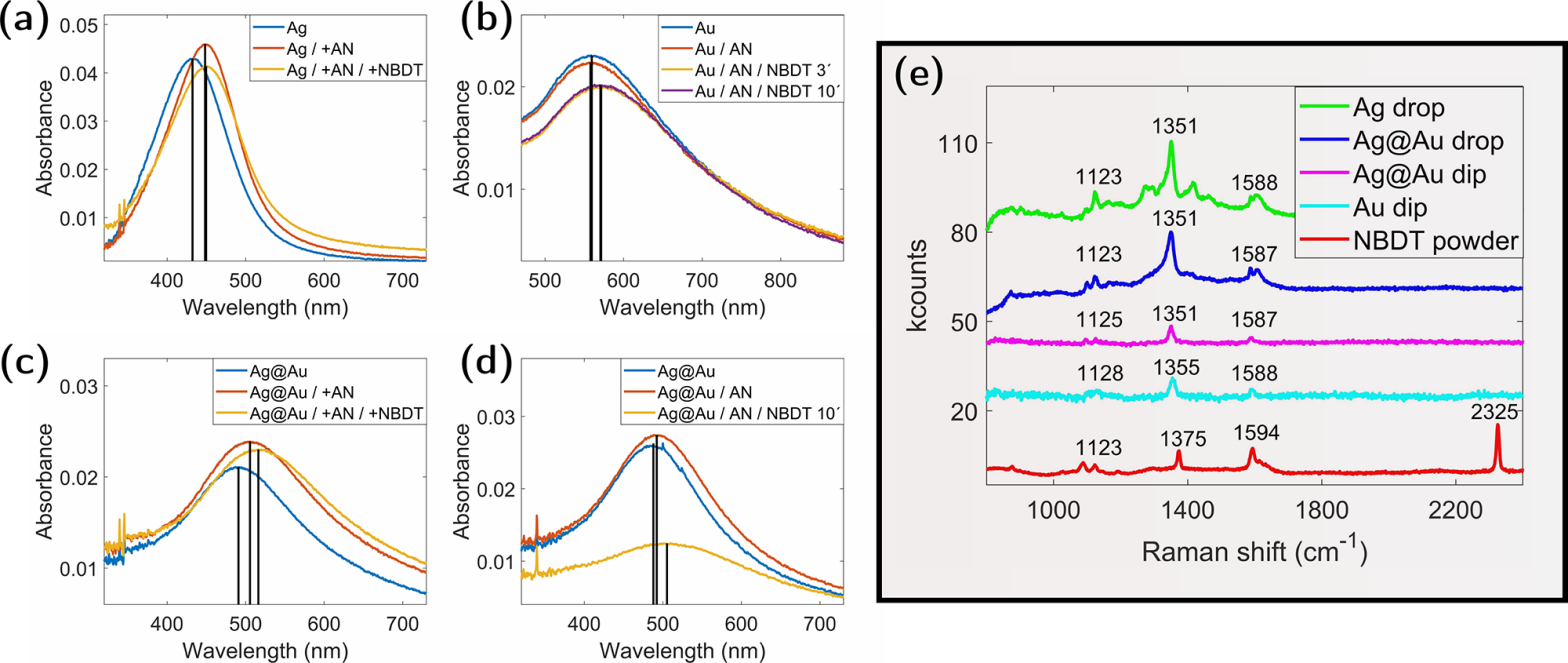
(a) UV/vis absorption spectra of a sample spot with silver nanoparticles treated with the drop procedure, the blue, red, and yellow curve correspond to the untreated, acetonitrile, and NBDT treated spot, respectively. (b) UV/vis absorption spectra of a gold nanoparticle sample treated with the dip procedure, the blue, red, yellow, and purple curve correspond to the untreated, acetonitrile, 3 min NBDT and 10 min NBDT-treated spot, respectively. (c) Same as (a) for silver@gold core@shell particles. (d) Same as (b) for silver@gold core@shell particles with only one 10 min treatment in the NBDT solution. (e) Raman spectra of the four deposition spots from panels (a–d) compared with a Raman spectrum measured for pure NBDT powder (red curve).

**Table 2:** Raman Shift (cm^–1^) of the Peaks from Figure 3 Compared with Literature Data for Nitrobenzene (NB)

Ag drop	Ag@Au drop	Ag@Au dip	Au dip	NB^54^	NBDT pow.	Raman mode
1123	1123	1125	1128	1122	1123	C–H bending
1351	1351	1351	1355	1348	1375	N=O stretching
1588	1587	1587	1588	1588	1594	C=C stretching
					2325	N≡N stretching

Finally, a significantly higher Raman signal yield is observed for the drop-prepared samples compared to the samples prepared by employing the dip procedure. This suggests that a higher amount of molecules is situated at SERS-hotspots in the case of the drop-prepared samples. This conclusion is supported by the significantly better resolution of the weaker Raman peaks in the spectra obtained from the samples prepared by the dropwise approach.

## Conclusions and Outlook

This study demonstrates that the presence of liquid bridges alters the arrangement of silver nanoparticles on SERS substrate surfaces. By exploiting this effect, we can simultaneously generate SERS hotspots and selectively equip them with Raman reporter molecules. The functionalization of nanoparticles composed of gold and silver with a diazonium salt, as explored in this work, is particularly significant, as for silver, no prior dediazoniation method has been reported for the selective and controlled placement of molecules at SERS hotspots. Owing to the strong plasmonic field enhancement of silver nanoparticles and the efficient binding of organic molecules to metal surfaces via dediazoniation, the investigated system offers substantial potential for the development of advanced SERS tags. These findings may contribute to expanding the repertoire of available SERS tags and broadening the areas of possible applications.
